# A Comparative Analysis of International Classification Systems to Predict the Risk of Collapse in Single-Level Osteoporotic Vertebral Fractures

**DOI:** 10.3390/diagnostics14192152

**Published:** 2024-09-27

**Authors:** Antonio Jesús Láinez Ramos-Bossini, Paula María Jiménez Gutiérrez, David Luengo Gómez, Mario Rivera Izquierdo, José Manuel Benítez, Fernando Ruiz Santiago

**Affiliations:** 1 Department of Musculoskeletal Radiology, Hospital Universitario Virgen de las Nieves, 18014 Granada, Spain; ajbossini@ugr.es (A.J.L.R.-B.); davidluengog@gmail.com (D.L.G.); ferusan12@gmail.com (F.R.S.); 2 Advanced Medical Imaging Group, Instituto Biosanitario de Granada (ibs.GRANADA), 18014 Granada, Spain; j.m.benitez@decsai.ugr.es; 3 Department of Anesthesiology, Hospital Universitario Virgen de las Nieves, 18014 Granada, Spain; 4 Department of Preventive Medicine and Public Health, University of Granada, 18015 Granada, Spain; mariorivera@ugr.es; 5 Department of Computer Science and Artificial Intelligence, University of Granada, 18016 Granada, Spain; 6 Department of Radiology and Physical Medicine, University of Granada, 18016 Granada, Spain

**Keywords:** fracture, spine, osteoporosis, vertebra, collapse, prognosis, classification, AO spine, imaging, radiology

## Abstract

Introduction: Various classifications for osteoporotic vertebral fractures (OVFs) have been introduced to enhance patient care and facilitate clinical communication. However, there is limited evidence of their effectiveness in predicting vertebral collapse, and very few studies have compared this association across different classification systems. This study aims to investigate the association between OVF categories, according to the most widely used classification systems, and vertebral collapse. Patients and Methods: A retrospective single-center study was conducted involving patients diagnosed with acute OVFs at the emergency department of a tertiary-level academic hospital with a minimum follow-up of 6 months. Vertebral fractures were independently classified by two radiologists according to several classification systems, including those proposed by Genant, Sugita, the German Society for Orthopedics and Trauma (DGOU), and the AO Spine. Associations between vertebral collapse and OVF classification systems were analyzed using bivariate and logistic regression analyses. Results: This study included 208 patients (82.7% females; mean age of 72.6 ± 9.2 years). The median follow-up time was 15 months, with L1 being the most common fracture site (47.6%). The most frequent OVF types observed, according to Genant’s morphological, Genant’s quantitative, Sugita ’s, DGOU’s, and AO Spine’s classifications, were biconcave (50%), grade 0.5 (47.6%), bow-shaped (61.5%), OF2 (74%), and A1 (61.5%), respectively. All classifications, except for Genant’s quantitative system, were significantly associated with vertebral collapse in bivariate analyses. Logistic regression analyses showed a significant association (*p* = 0.002) between the AO Spine classification and vertebral collapse, with 85.7% of A4 fractures developing collapse on follow-up. Conclusions: The AO Spine classification showed the highest predictive capacity for vertebral collapse. Specifically, A4 fracture types showed a very high risk of vertebral collapse, confirming the need for non-conservative management of these fractures. Further multicentric and prospective studies are warranted to confirm these findings.

## 1. Introduction

Osteoporotic vertebral fractures (OVFs) represent the most frequently occurring type of osteoporotic fracture and are associated with high morbidity and mortality [1,2]. Vertebral collapse, usually defined as a significant loss of vertebral height (>50%), is a potential end-stage evolution of OVFs [3]. It is associated with increased kyphotic deformity, pain, and fracture site instability, potentially affecting patient management [4]. Therefore, preventing vertebral collapse is important for clinical practice.

A number of factors have been identified as potential predictors of vertebral collapse, including middle column injury, burst fractures, location in the thoracolumbar transition (T12-L1), and ages over 50 years [5,6]. However, they are not completely understood, and a knowledge gap still exists. Probably the most relevant approaches are based on the identification of biomarkers associated with imaging parameters obtained in X-rays, computed tomography (CT), or magnetic resonance imaging (MRI). Different classification systems have been developed with the aim of characterizing and predicting which OVFs could require specific therapeutic approaches to prevent the progressive loss of vertebral height and eventual collapse, which can be consulted in more detail in previous studies [7].

For example, the semiquantitative Genant et al.’s classification [8] categorizes vertebral fractures based on the morphology and degree of area and height loss. Sugita et al. classified fractures into 5 morphological types based on radiographic findings, with swelled, bow-shaped, and projecting types being frequently associated with the presence of intravertebral cleft and late collapse, as well as with a worse prognosis [9]. The German Society for Orthopedics and Trauma (DGOU) proposed a classification system for OVFs that offers a comprehensive score based on the type of fracture and clinical factors. Initial results suggest that this score is an appropriate tool for the preoperative assessment of OVFs [10]. Similarly, the AO Spine classification system [10] developed a comprehensive classification system, including specific categories for typical OVFs (A category), which shows a high interobserver agreement [11].

However, there is no universal agreement on which of these systems is the best to guide patient management. Some authors have pointed out the need to further explore the role of available classifications in predicting fracture evolution, with particular emphasis on their role in specific therapies, such as percutaneous vertebroplasty [12]. However, the evidence is still scarce in this context, and factors such as the incidence of different types of OVFs and collapse, according to each classification system, are required.

The aim of this study is to explore the association between the most widely used classification systems for OVFs and the development of vertebral collapse. 

## 2. Materials and Methods

### 2.1. Study Design and Participants

We conducted a retrospective single-center study based on a consecutive series of patients diagnosed with acute OVFs in our institution, which is a tertiary hospital specializing in traumatology that attends to patients from 3 secondary hospitals, from January 2019 to December 2022. This study was approved by our Institutional Review Board (code TFG-FX-2019). The Strengthening the Reporting of Observational Studies in Epidemiology (STROBE) guidelines [13] were followed when designing and reporting this study.

### 2.2. Inclusion and Exclusion Criteria

We performed a systematic search in our radiology information system, including all thoracic and lumbar spinal X-rays and CTs performed during the study period, with the keywords ‘vertebr*’ and ‘fracture’. All reports were read by two independent researchers, who did not participate in the radiological assessment of fractures, and medical records of patients meeting radiological criteria were revised. 

The following inclusion criteria were established:

1. Patients diagnosed with acute OVF, identified on X-rays and CT.
2. Patients with imaging follow-up of their fracture at least 6 months after diagnosis.
3. Management with conservative medical treatment.

The exclusion criteria were as follows:

1. Genant et al.’s grade 3 (loss of height/area > 40%) OVFs at initial diagnosis.
2. More than 1 acute OVF.
3. Patients treated by surgery or vertebral augmentation.
4. Patients with poor-quality images, such as rotated, non-parallel radiographs.

### 2.3. Variables of the Study

Sociodemographic data (age and sex), the OVF location, and cause were obtained. The dependent variable was vertebral collapse, defined as the presence of >50% loss of vertebral body area or height at the end of follow-up. All fractures at initial diagnosis and follow-up were independently classified by 2 different radiologists, according to the classification systems proposed by Genant et al. (morphological and quantitative, including grade 0.5 for OVFs with 20% loss of vertebral height/area) [8], Sugita et al. [9], the German Society for Orthopaedics and Trauma [13], and the AO Spine [14]. In case of a disagreement, the case was revised and discussed until an agreement was reached.

### 2.4. Statistical Analysis

We carried out a descriptive analysis of the measured variables by expressing qualitative variables as absolute (*n*) and relative (%) frequencies, and numerical data as mean (X) and standard deviation (SD). Next, bivariate analyses were performed to explore the association between fracture type, according to each classification system, and vertebral collapse, using chi-squared tests (or Fisher’s exact tests, if the data did not meet statistical assumptions for parametric tests). Then, logistic regression analyses, to predict the dependent variable ‘vertebral collapse’, were applied to the variables that were statistically significant in bivariate analyses. In these analyses, we processed categorical variables by comparing each category to a reference category, which provided a single coefficient that summarized the overall effect of the variable on the odds of vertebral collapse. For the classification systems that resulted significance, we calculated the risk of vertebral collapse for each vertebral fracture category using odds ratios. Finally, receiver operating characteristic (ROC) curve analysis for the classification systems significantly associated with the primary outcome was performed.

Two-tailed tests were performed with significant values set at *p* < 0.05. All statistical analyses were carried out with R software version 4.3.2 for Windows (Vienna, Austria) [15].

## 3. Results

### 3.1. Characteristics of the Sample

The final sample included a total of 208 patients (82.7% women; mean age, 72.8 ± 9.2 years). The median follow-up time was 14.5 months (interquartile range, 14.5 months). The most frequent cause of the fracture was falling from standing height, which occurred in 190 cases (91.3%), followed by spontaneity or exertion (4.3% each). The most frequently fractured vertebral body was L1 (48.3%), followed by T12 and L2 (15% each). Vertebral collapse was observed in 85 cases (40.9%).

### 3.2. Classification of Osteoporotic Vertebral Fractures and Association with Collapse

Regarding the incidence of different types of OVFs according to each classification, the most frequent ones, according to Genant et al.’s morphological classification, were biconcave fractures (50%), and the most prevalent quantitative grading category was grade 0.5 (47.6%). Regarding Sugita et al.’s classification, the most frequent type was bow-shaped (61.5%). For the AO Spine classification, the most frequent fracture type was A1 (61.5%). Finally, according to the DGOU classification, the most frequent OVF type was OF2 (74%). 

**Figure 1:**
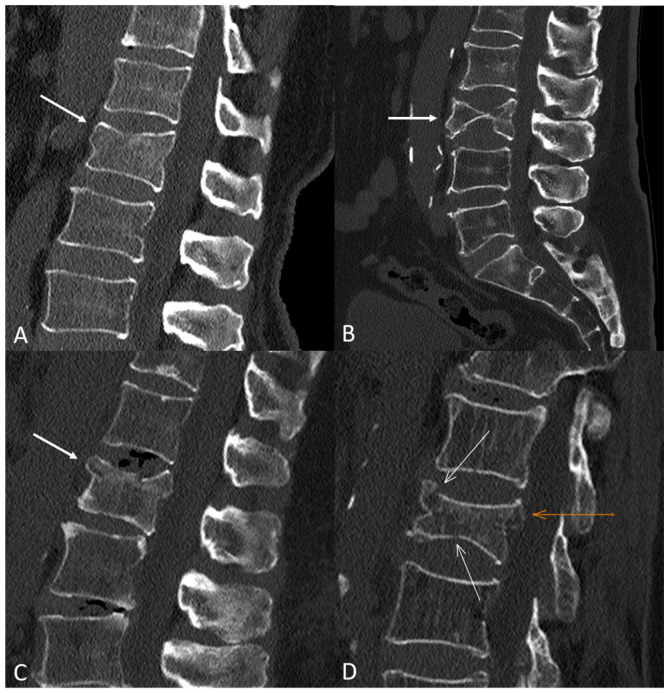
Illustrative examples of osteoporotic vertebral fractures in our sample based on the AO Spine classification system. (**A**) A1-type fracture of L1 (white arrow). Note the subtle loss of vertebral height and the trabecular fracture line below the superior endplate, as well as the absence of posterior wall displacement. (**B**) A2-type fracture of L3 (white arrow) with a typical pincer-like morphology, involving the central part of the vertebral body. (**C**) A3-type fracture of L1 (white arrow) involving the superior endplate, with mild displacement of the posterosuperior wall to the spinal canal. (**D**) A4-type fracture of L2, with involvement of the superior and inferior endplates (white arrows) and displacement of the posterosuperior wall to the spinal canal (orange arrow).

**Figure 2:**
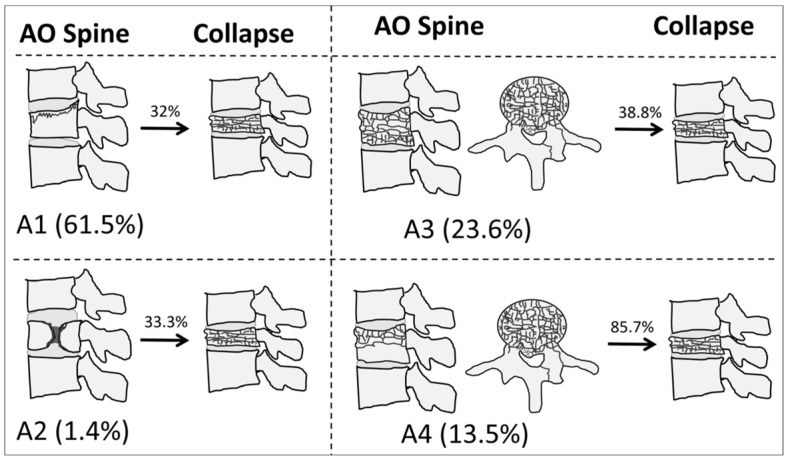
The incidence and rate of collapse of osteoporotic vertebral fractures, according to the AO Spine classification system in our sample.

Bivariate analyses showed significant associations between all classification systems, except Genant et al.’s quantitative, and vertebral collapse. The highest rate of collapse among all classifications was observed in the A4 category of the AO Spine, with 24 out of 28 (85.7%) OVFs developing vertebral collapse on follow-up. Figure 1 shows illustrative examples of A1-A4 AO Spine classification fractures, and Figure 2 summarizes the incidence of collapse in these fractures. Table 1 shows the incidence of the different types of OVFs and the results of bivariate analyses.

### 3.3. Prediction of Vertebral Collapse: Regression and ROC Curve Analyses

Univariate binary logistic regression analyses, including the classifications that were associated with vertebral collapse in bivariate analyses, showed statistically significant associations for the AO Spine (*p* < 0.001) and the DGOU (*p* = 0.007) classifications. Multivariate regression analysis, including both classifications, was significant only for the AO Spine classification (*p* = 0.001). A trend toward significance was observed in the DGOU classification (*p* = 0.121) (Table 2). Regression analyses of the categories of the AO Spine classification showed statistically significant associations for A4 (*p* < 0.001; OR, 12.73 [95%CI, 4.57–45.47]) (Table 3). The rest of the fracture categories were not significantly associated with vertebral collapse.

**Figure 3:**
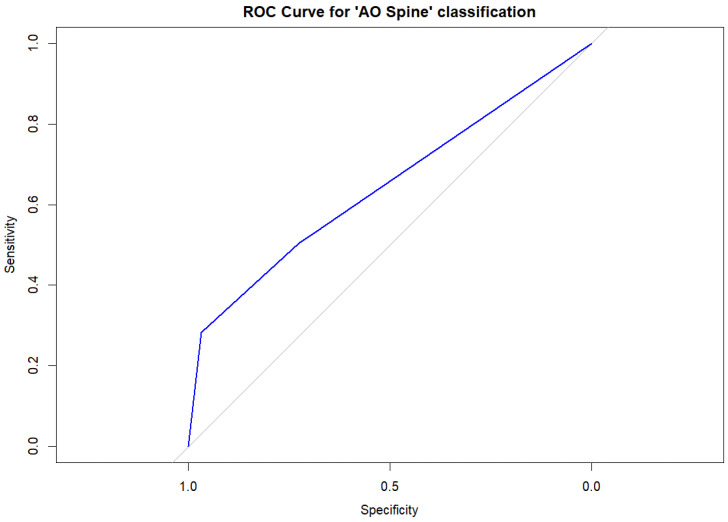
Receiver operating characteristic (ROC) curve analysis for the AO Spine classification in our sample. The blue line represents the ROC curve based on the logistic regression results, where the AO Spine classification was the only significant predictive variable. The gray diagonal line corresponds to the reference of a random classification (line of no discrimination).

The ROC curve analysis of the AO Spine classification to predict vertebral collapse showed an area under the curve (AUC) of 0.646 (95%CI, 0.567–0.725), mainly explained by the effect of the A4 fracture type (AUC, 0.625, 95%CI, 0.574–0.676). For this category, the sensitivity and specificity for vertebral collapse were 0.282 and 0.967, respectively. Figure 3 shows the ROC curve for the AO Spine classification.

## 4. Discussion

Our results provide interesting insights regarding the epidemiology and status quo of conservatively managed OVFs and their classifications. First of all, the epidemiological data found are consistent with previous studies; there was an evident female predominance (82.7%) of old age (median: 72.8 years), and the most frequent fractured vertebral body levels were T12 and L1, as in previous studies [16,17,18]. Interestingly, we observed a high incidence of vertebral collapse (40.9%) in the sample. This indicates that conservative management was of limited value in avoiding poor fracture evolution in OVFs with the characteristics defined in our inclusion criteria. However, it should be noted that the criteria used to define failure of conservative treatment are heterogeneous in the literature, as pointed out in the systematic review by Petitt et al. [19]. Notably, a relatively high number (37%) of burst (AO Spine A3 and A4) fractures were observed in our sample, which may explain the fact that the incidence of vertebral collapse was significantly higher compared to previous studies. For instance, Funayama et al. reported 20.9% collapse in a cohort of patients with OVFs managed conservatively with bed rest [20], and a similar figure to that was reported by Kanchiku et al. (16 + 11%) [21]. 

In addition, the distribution of the types of OVFs, according to different classification systems, offers important information. Regarding Genant et al.’s classification, we found that the most frequent morphological types were biconcave fractures (50%), and the most prevalent quantitative grading was grade 0.5 (48.3%). It should be noted that we excluded grade 3 OVFs, since the loss of vertebral height in this category (>50%) is almost in the range of collapse. For the AO Spine classification, the most frequent fracture type was A1 (56.7%). Notably, there were no A0 fractures, and only 3 cases of A2 category were found. These results are similar to those of previous studies [22]. We hypothesize that the absence of A0 fractures may be due to the fact that they are more subtle and could have been missed in the original reports—note that we performed a retrospective study on the radiological information system through keywords—or even confused for non-fused transverse processes. In any case, these fractures are managed conservatively and straightforwardly, as they do not affect segmental stability [23]. On the other hand, the low number of A2 fractures (only 3) found in our sample should be considered when interpreting our results, as it limits the reliability of the prognostic models used on the AO Spine fracture subtypes. Finally, according to the DGOU classification, the most frequent type was OF2 (75%).

Third, we found that, among the different classification systems explored, only the one proposed by the AO Spine was associated with vertebral collapse. In particular, approximately two thirds of A1 fractures did not collapse. Conversely, 24 out of 28 A4 fractures collapsed in our series, while A3 fractures were very similar in both groups (collapse, 30.8%; non-collapse, 32.4%). The odds ratio for vertebral collapse in A4 vertebral fractures (using A1 as the reference standard) was 15.4, although the confidence interval was ample due to the relatively limited data available for multinomial variables in our sample. The AUC of the model for AO Spine fractures was moderate (0.646) and particularly influenced by the A4 category, with poor sensitivity (28.2%) and high specificity (96.7%), indicating that the model helps predict those fractures which will not collapse. Notably, this model only takes into account the predictive power of the AO Spine classification, which could be significantly increased with multivariate models that should be explored in future studies [3].

Theoretically, both A3 and A4 types are considered unstable fractures, and should thus be managed surgically or with vertebral augmentation techniques. This is in agreement with previous reports showing a tendency to surgically manage A3/A4 OVFs [24,25,26]. However, our results show a neat difference in their prognosis (along with their incidence). This emphasizes the difficulty of choosing an appropriate and standardized treatment in osteoporotic burst fractures (i.e., A3 and A4). In addition, the surgical approach to A3 and A4 fractures tends to differ in real-world practice, with A4 fractures more frequently being operated through anterior approaches. Moreover, A3 fractures present with worse ASIA Impairment-Scale scores at admission, compared to A4 fractures [27].

Regarding other classification systems, in the Sugita et al.’s classification, the most frequent category observed was bow-shaped (60%). Interestingly, although Sugita et al. suggested that swelled-front, bow-shaped, and projecting types had a higher risk of collapse [9], our findings only partially support the idea that the swelled-front type may be associated with a higher risk of collapse, as it was present in 62.5% of fractures that eventually collapsed compared to 37.5% of non-collapse OVFs. For bow-shaped formations, we observed contrary results, as these were much more frequent in OVFs that collapsed (68.8% vs. 31.3%), and similar frequencies were observed in the case of projecting fractures (46.2% and 53.8%). Finally, the fact that the DGOU classification showed a trend toward significance for its association with vertebral collapse should also be noted. Nevertheless, none of the categories were significantly associated with vertebral collapse, although incidence data might suggest that OF1 and OF2 fractures have a lower tendency to collapse.

Further studies should explore the particular role of associated factors with a predictive capacity for vertebral collapse. A previous study by Pazos Mohri et al. found that immediate standing collapse of thoracolumbar burst OVFs predicts final vertebral collapse in patients [28]. It is plausible that, with the introduction of novel diagnostic approaches, specific factors associated with vertebral collapse will be identified in the near future. For instance, a recent study by Cho et al. [29], using advanced statistics and machine learning approaches, reported that the initial shape of the vertebra could predict progressive collapse in OVFs. Similarly, fractal analysis in CT and MRI images, which evaluates the complexity of bone microarchitecture, could offer further details about the structural weaknesses that predispose certain fractures to collapse [30,31]. The potential of fractal analysis to complement existing classification systems for vertebral fractures makes it a valuable area for future research.

The limitations of this study include its retrospective, single-center nature, and the relatively limited sample size, especially considering the multinomial nature, and the disbalanced categories of the classification systems. In addition, we only explored the role of classification systems for OVFs, but, as previous studies have highlighted [5,32,33], a number of factors beyond the type of fracture need to be considered to standardize the most appropriate treatment. Therefore, future studies, ideally prospective and multicentric, should confirm our findings and correlate them with other variables that could influence the prognosis of OVFs.

## 5. Conclusions

The AO Spine is the most useful classification system to predict vertebral collapse in osteoporotic vertebral fractures. Specifically, A4 fractures were associated with a high risk of vertebral collapse, confirming the need for non-conservative management in these fractures. Further multicentric and prospective studies are warranted to confirm these findings and refine treatment guidelines.

## Figures and Tables

**Table 1:** Univariate and bivariate analyses of the types of osteoporotic vertebral fractures, according to different classification systems in our sample. ^1^ Data are expressed as absolute (*n*) and relative (%) frequencies. * Statistically significant *p*-value.

Type of Fracture	Total Sample *n* (%) ^1^	No Collapse (*n* = 85) *n* (%)	Collapse (*n* = 143) *n* (%)	*p*-Value
Genant (morphological)		a	a	
Wedge	91 (43.8)	54 (51.9)	50 (48.1)	0.01 *
Biconcave	104 (50)	64 (70.3)	27 (29.7)	
Crush	13 (6.3)	5 (38.5)	8 (61.5)	
Genant (semiquantitative grading)				
Grade 0.5	99 (47.6)	58 (58.6)	41 (41.4)	0.843
Grade 1	46 (22.1)	26 (56.5)	20 (43.5)	
Grade 2	63 (30.3)	39 (61.9)	24 (38.1)	
Sugita				
Swelled-front	32 (15.4)	12 (37.5)	20 (62.5)	0.008 *
Bow-shaped	128 (61.5)	88 (68.8)	40 (31.3)	
Projecting	26 (12.5)	12 (46.2)	14 (53.8)	
Concave	11 (5.3)	5 (45.5)	6 (54.5)	
Dented	11 (5.3)	6 (54.5)	5 (45.5)	
AO Spine				
A1	128 (61.5)	87 (68)	41 (32)	<0.001 *
A2	3 (1.4)	2 (66.7)	1 (33.3)	
A3	49 (23.6)	30 (61.2)	19 (38.8)	
A4	28 (13.5)	4 (14.3)	24 (85.7)	
DGOU				
OF1	9 (4.3)	7 (77.8)	2 (22.2)	0.049 *
OF2	154 (74)	97 (63)	57 (37)	
OF3	39 (18.8)	17 (43.6)	22 (56.4)	
OF4	6 (2.9)	2 (33.3)	4 (66.7)	

**Table 2:** Univariate and multivariate logistic regression analysis for the outcome “vertebral collapse”. OR, odds ratio. aOR, adjusted odds ratio.

Classification System	*p*-Value [Univariate]	OR [95% CI]	*p*-Value [Multivariate]	aOR [95%CI]
Sugita	0.852	1.024 [0.800–1.309]	-	-
Genant (morphological)	0.266	0.770 [0.485–1.221]	-	-
Genant (quantitative)	0.709	0.941 [0.683–1.296]	-	-
AO Spine	<0.001	1.418 [1.192–1.686]	0.001	1.356 [1.130–1.626]
DGOU	0.007	2.059 [1.215–3.490]	0.121	1.545 [0.891–2.679]

**Table 3:** Odds ratios (OR) for the AO Spine classification fracture categories and vertebral collapse. ^1^ Ref, reference category (the one with the highest frequency was chosen). * Statistically significant *p*-value. 95% confidence interval (CI).

AO Spine [Ref ^1^, A1]	Estimate	Std. Error	*p*-Value	OR [95%CI]
Intercept	−0.752	0.189	<0.001 *	0.47 [0.32–0.68]
A2	0.059	1.239	0.926	1.06 [0.05–11.38]
A3	0.296	0.349	0.397	1.34 [0.67–2.65]
A4	2.544	0.572	<0.001 *	12.73 [4.57–45.47]

## Data Availability

The data presented in this study are available on request from the corresponding author due to restrictions imposed by the Ethics Committee which approved the study protocol.
